# Decision-Making in COVID-19 and Frailty

**DOI:** 10.3390/geriatrics5020030

**Published:** 2020-05-06

**Authors:** Susan Moug, Ben Carter, Phyo Kyaw Myint, Jonathan Hewitt, Kathryn McCarthy, Lyndsay Pearce

**Affiliations:** 1 Department of Surgery, Royal Alexandra Hospital, Paisley PA2 9PN, UK; 2 School of Medicine, Dentistry and Nursing, University of Glasgow, Glasgow G12 8QQ, UK; 3 Trial Statistician, Kings College London, London WC2R 2LS, UK; ben.carter@kcl.ac.uk; 4 Institute of Applied Health Sciences, University of Aberdeen, Aberdeen AB25 2ZD, UK; phyo.myint@abdn.ac.uk; 5 Department of Population Medicine, Cardiff University, Cardiff CF10 3XQ, UK; hewittj2@cardiff.ac.uk; 6 Department of Surgery, North Bristol NHS Trust, Southmead Rd, Bristol BS10 5NB, UK; drkathrynmccarthy@hotmail.co.uk; 7 Department of Surgery, Salford Royal NHS Foundation Trust, Stott Ln, Salford M6 8HD, UK; Lyndsay.pearce@srft.nhs.uk

We write in response to the COVID-19 pandemic and the important recognition of co-existing frailty [COVID-19 rapid guideline: critical care in adults; NICE NG159] [1]. There is no doubt that difficult decisions have been made and are continuing to be made across the UK. These decisions will become increasingly difficult with the continued narrowing of the clinical criteria for the escalation of treatment, as has been seen in other countries globally. Frailty has been placed at the forefront, with professional associations responding to the NICE NG159 by proposing a frailty score as part of the clinical assessment [2]. There is no doubt that increasing frailty (irrespective of the scale or score applied) is associated with poorer outcomes in both medical and surgical patients, with increased ITU admissions, prolonged length of stay, increased care needs on discharge and mortality all reported [3,4]. However, these studies were not performed during a viral pandemic and while we, like all clinicians, await published evidence on COVID-19, we would like to highlight some points to consider when making clinical decisions based on frailty [5].

## 1. Frailty Is a Spectrum and Is Not a Binary Phenomenon

When using frailty scores, clinicians should be familiar with how to score patients accurately. The subtleties of difference between Clinical Frailty Scores may identify patients to be frail (Clinical Frailty Score 5) rather than vulnerable (CFS 4) and as such impact their treatment or therapy options. Clinicians should also be aware of the different scores and how to apply them [this includes the two different Clinical Frailty Scores (1–7 and 1–9)]. Many societies provide training modules online [2].

## 2. Younger Adults May Be Frail, Whilst Older Adults May Not Be

Frailty has been shown to be independent of age when predicting poorer outcomes [4]. As a result, clinicians should be aware that frailty is not a certainty in older adults and that younger adults (those under 65 years of age) can be frail too [6]. Assumptions should not be made solely on age.

## 3. Frailty Does Not Define Futility 

This is where the grey area lies, and where shared decision-making with patients, relatives, carers, and other and/or experienced clinicians is vital. The additional challenges of limited ‘face to face’ discussions of this nature for COVID-19 patients and their next of kin cannot be underestimated. 

We do not aim to confuse or judge clinical decision-making. We simply want to highlight that frailty is not perfect in helping decisions to be made, but it is excellent at highlighting risk, which can prompt discussion about escalation of treatment, mortality, patient wishes and DNACPR. 

NICE include a template for a Decision-Making Form which we fully endorse. Completion of such forms should be considered for all stages of hospital admission (ward/surgery/critical care/DNACPR) with the involvement of a ‘new decision-making MDT’. Such a team could include critical care, anaesthetics, surgeons, medics, palliative care, geriatricians and nursing ward staff, and will provide patients with balanced viewpoints, as there can be variation in clinician-estimates of survival. Not all of these specialties need to participate in every decision, but instead should be available to be included to spread the burden of making frequent life-changing decisions. This will provide vital support for clinicians in an area that is increasingly going to form part of their routine working day.
